# Treatment of Syphilis*Abridged from L’Experience, No. 141; and London Lancet, April 11, 1840, p. 112.

**Published:** 1840-07

**Authors:** 


*Treatment of Syphilis.**—M. Ricord has recently made some observations on the treatment of syphilis, in a French journal, of which the following is the substance:

M. Ricord divides the progress of syphilis into three stages or phases. In the first, the action of the virus is completely local; in the second stage, the accidents are confined to the skin, or mucous membranes, and are characterised by the fact that the morbid products are incapable of producing the original disease, or inoculation. The symptoms of the third stage rarely occur bofore the seventh month, and are incapable of being transmitted by hereditary disposition: this is their characteristic mark.

---

* Abridged from L'Experience, No. 141; and London Lancet, April 11, 1840, p. 112.

M. Ricord considers the mercurial treatment to be more frequently injurious than useful in the first stage. On the contrary, mercury is absolutely necessary in the second stage.

Where the tertiary symptoms alone exist, M. Ricord has generally recourse to the ioduret of potassium. He begins with doses of ten grains in the following manner:

Distilled water, 3 ℥;
Ioduret of potassium, 10 grs.;
Syrup of poppies, 1 ℥.

This potion is taken in three doses, during the day, with sarsaparilla, the quantity of the ioduret gradually increased every five days, until the patient takes 100 grains a day.

Whenever secondary symptoms coexist with the tertiary, M. Ricord administers the proto-ioduret of mercury in the dose of a grain, gradually increased to six grains.—*American Med. Lib.*

